# Is Nitrogen a Key Determinant of Water Transport and Photosynthesis in Higher Plants Upon Drought Stress?

**DOI:** 10.3389/fpls.2018.01143

**Published:** 2018-08-22

**Authors:** Lei Ding, Zhifeng Lu, Limin Gao, Shiwei Guo, Qirong Shen

**Affiliations:** ^1^Jiangsu Provincial Key Lab for Organic Solid Waste Utilization, National Engineering Research Center for Organic-based Fertilizers, Jiangsu Collaborative Innovation Center for Solid Organic Waste Resource Utilization, Nanjing Agricultural University, Nanjing, China; ^2^Louvain Institute of Biomolecular Science and Technology, Université catholique de Louvain, Louvain-la-Neuve, Belgium

**Keywords:** drought stress, water transport, photosynthesis, nitrogen, aquaporin

## Abstract

Drought stress is a major global issue limiting agricultural productivity. Plants respond to drought stress through a series of physiological, cellular, and molecular changes for survival. The regulation of water transport and photosynthesis play crucial roles in improving plants’ drought tolerance. Nitrogen (N, ammonium and nitrate) is an essential macronutrient for plants, and it can affect many aspects of plant growth and metabolic pathways, including water relations and photosynthesis. This review focuses on how drought stress affects water transport and photosynthesis, including the regulation of hydraulic conductance, aquaporin expression, and photosynthesis. It also discusses the cross talk between N, water transport, and drought stress in higher plants.

## Introduction

Crop production is facing threats from both biotic and abiotic stresses. Drought stress is considered to be one of the most devastating abiotic stresses, and it decreases crop yield, particularly in arid and semiarid areas (Chaves et al., 2003; Parry et al., 2007; Lambers et al., 2008). The decrease in yield varies from 13 to 94% in the investigated crops that were under drought stress (Farooq et al., 2009). Rice is traditionally cultivated in waterlogged conditions, and in China, 80% of the freshwater used in agriculture is for rice production, indicating that rice production would suffer more drought stress due to water shortages (Guo et al., 2007a). It is expected that drought stress would be more severe because of global warming (Chang, 2007).

In higher plants, drought stress induces an array of physiological and biochemical adaptations of metabolism for survival by increasing the drought resistance through three strategies, namely, “drought escape,” “drought avoidance,” and “drought tolerance” (Morgan, 1984; Xu et al., 2010; Vilagrosa et al., 2012). Strategies of drought escape include reducing life span and inducing vegetative dormancy to escape severe drought stress (Geber and Dawson, 1990; Vilagrosa et al., 2012). Strategies of drought avoidance include increasing water uptake ability and water use efficiency, for example, stomatal closure, extensive root systems, high capacity for water transport from roots to leaves, and high leaf mass to leaf area ratio (Schulze, 1986; Jackson et al., 2000). Strategies of drought tolerance mainly include improving osmotic adjustment ability, increasing cell wall elasticity to maintain tissue turgidity, increasing antioxidant metabolism, increasing compatible solutes, and enhancing the resistance to xylem cavitation (Morgan, 1984).

In this review, we present an overview on how drought stress affects water uptake, transport, and photosynthesis in higher plants. In particular, we summarize that nitrogen (N) supply may regulate drought tolerance in higher plants with different N forms and/or N levels. Nitrogen is an essential macronutrient for plants, and it can affect many aspects of plant growth and metabolic pathways (Guo et al., 2007b; Xu et al., 2012; Wang et al., 2014). Ammonium and nitrate are two major N sources in higher plants. It is well-documented that these N forms regulate drought tolerance through root water uptake and photosynthesis in rice (Li et al., 2009a, 2012; Yang et al., 2012; Ding et al., 2016b), French beans (Guo et al., 2002, 2007b), and maize (Mihailović et al., 1992).

## Drought Stress Affects Water Uptake and Transport

In soil-plant-atmosphere continuum system, water travels from soil to the atmosphere. Two water flow pathways are included in this process: axial movement (water flow from root xylem to leaf vessels) and radial movement (water flow from soil to root xylem and from leaf xylem vessels to mesophyll cells) (Sade and Moshelion, 2017). The whole plant hydraulic conductance is determined by radial conductance, that is, root hydraulic conductivity (Lpr) and leaf hydraulic conductance (K_leaf_), since water must pass through apoplastic barriers, which resist the water flow (Steudle and Peterson, 1998; Sack and Holbrook, 2006). During drought stress, both Lpr and K_leaf_ are affected in higher plants (Aroca and Ruiz-Lozano, 2012; Sade and Moshelion, 2017).

### Drought Stress Affects Lpr and K_leaf_

Root hydraulic conductivity tends to decrease during drought stress (North et al., 2004; Aroca et al., 2012; Grondin et al., 2016; Meng and Fricke, 2017). The decrease in Lpr (1) causes a decrease in transpiration and an increase in water use efficiency (Iuchi et al., 2001) and (2) evades water leakage from root back into soil while soil water content decreases progressively (Jackson et al., 2000). Nonetheless, an increase in Lpr was observed after short-term water stress treatment with polyethylene glycol (PEG) 6000 in rice (Ding et al., 2016b) and maize (Hose et al., 2000). In other studies, decrease in Lpr was detected after short-term water stress treatment (with PEG) in cucumber (Qian et al., 2015) and tobacco (Mahdieh et al., 2008). The response of Lpr to drought stress varies among species, indicating that there are different strategies for water uptake regulation. It can be seen that water distribution is non-uniform when the soil becomes dry. McLean et al. (2011) demonstrated that one half of the roots increased the capacity of water uptake in a wet zone, whereas the other half of the roots decreased water uptake in a dry zone.

Vandeleur et al. (2009) showed that, in grapevine under drought stress, Lpr decreased while cell hydraulic conductivity (Lpc) increased. Similar result was obtained by Hachez et al. (2012) in maize, and it was demonstrated that Lpc increased after 2 h of PEG treatment, without any further change in Lpr. Such an increase of Lpc might be helpful for osmotic adjustment. It was postulated that Lpr was controlled by the conductivity of exo- and endodermis cells, while not cortical cells (Lpc) under water stress, since large resistance was expected for water flow passing exo- and endodermis due to the deposition of lignin and suberine in these cells (Hachez et al., 2012).

In leaves, drought stress induced the decrease of both leaf water potential (Ψ_leaf_) and K_leaf_ in many plants, including woody species (Johnson et al., 2009; Scoffoni et al., 2011a), grapevine (Pou et al., 2013), Arabidopsis (Shatil-Cohen et al., 2011), and sunflower (Nardini and Salleo, 2005). Water movement inside leaves includes two pathways (1) water movement through leaf xylem (i.e., petiole and venation) and (2) water movement outside the xylem (i.e., bundle sheath and mesophyll) (Sack and Holbrook, 2006). When plants suffer from drought stress, both water flow pathways are affected (Scoffoni et al., 2011b) and aquaporins play an important role in regulating water movement outside the xylem (Buckley, 2015). Decrease in K_leaf_ was associated with the downregulation of aquaporin expression and/or activity in bundle sheath cells under drought stress (Shatil-Cohen et al., 2011). Additionally, it was demonstrated that abscisic acid (ABA) accumulation inside leaves induced the downregulation of aquaporin activity in bundle sheath cells, which further induced the decrease of K_leaf_ under drought stress. Indeed, overexpressing the aquaporin gene (*NtAQP1*) in bundle sheath cells reduced the effect of ABA on K_leaf_ (Sade et al., 2015). On the other hand, leaf xylem embolism by cavitation formation decreased K_leaf_ under drought stress (Johnson et al., 2009; Scoffoni et al., 2011b; Vilagrosa et al., 2012).

### Drought Stress Affects Lpr Through the Regulation of Aquaporin

In the “composite transport model” (Steudle and Peterson, 1998; Steudle, 2000a), water flows from soil to root xylem in two parallel pathways, namely, apoplastic pathway and cell-to-cell pathway. Apoplastic water flow is blocked by apoplastic barriers in exodermis and endodermis, and the flow must proceed through the cell-to-cell pathway, which has large resistance for water movement (Maurel, 1997). Yet, aquaporins located on the membrane reduce the resistance. Aquaporins play an important role in regulating Lpr (Javot and Maurel, 2002; Gambetta et al., 2017). Vandeleur et al. (2014) showed that shoot topping decreased Lpr by 50–60%, through the downregulation of aquaporin gene expression (five to tenfold decrease). Gambetta et al. (2017) reviewed that the contribution of aquaporin to Lpr is highly variable across species, ranging from 0∼90%, and the variability depends on the type of aquaporin inhibitor and the method used to measure Lpr. Genetically modified aquaporin expression is used to change Lpr, which was decreased by 42% in *NtAQP1* knockouts, antisense tobacco plants deficient in the tobacco aquaporin NtAQP1, and by 20∼30% in *AtPIP1;2* knockouts, *Arabidopsis thaliana* plants deficient in the aquaporin AtPIP1;2 (Postaire et al., 2010).

**FIGURE 1:**
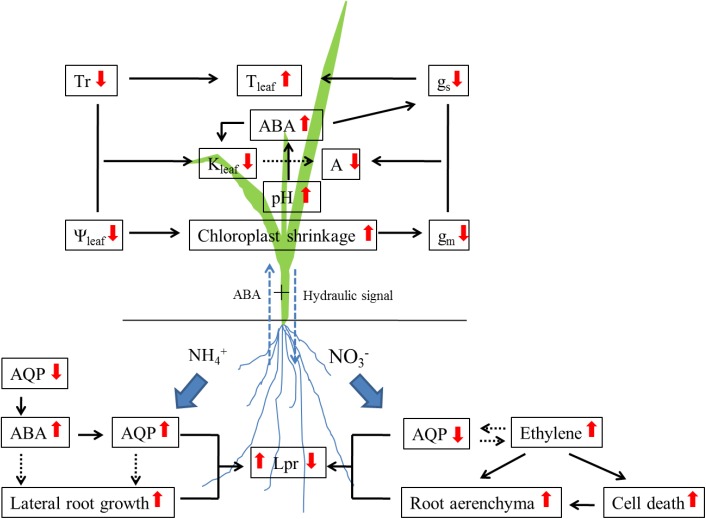
The mechanism of nitrogen form affecting drought tolerance in rice plants. NH_4_^+^, Ammonium; NO_3_^-^, Nitrate; AQP, Aquaporin; ABA, Abscisic acid; Lpr, Root hydraulic conductivity; Tr, Transpiration rate; Ψ_leaf,_ Leaf water potential; T_leaf,_ Leaf temperature; K_leaf,_ Leaf hydraulic conductance; A, Photosynthetic rate; g_s_, Stomatal conductance; g_m_, Mesophyll conductance. Up arrows, increase; down arrows, decrease.

Under drought stress, the change in Lpr is associated with the regulation of aquaporin expression (Steudle, 2000b; Aroca and Ruiz-Lozano, 2012; Aroca et al., 2012; Henry et al., 2012). The contribution of aquaporins to Lpr was up to 85% under drought stress in rice (Grondin et al., 2016). Four rice genotypes showed increased contribution, whereas two showed decreased contribution after long-term drought treatment in comparison with well-watered treatment. Our results demonstrated that ammonium nutrition enhanced drought tolerance in rice seedlings when compared with nitrate nutrition (Guo et al., 2007a; Li et al., 2009a), which is associated with the regulation of aquaporin expression (see **Figure 1**; Gao et al., 2010; Yang et al., 2012; Ding et al., 2015, 2016b). After 24 h of water stress treatment with PEG 6000, the expression and activity of aquaporins were enhanced in plants supplied with ammonium when compared with normal water treatment, whereas no increase was observed in plants supplied with nitrate (Ding et al., 2015, 2016b). Furthermore, it was observed that ABA accumulation was much faster in roots supplied with ammonium than with nitrate during 24 h drought treatment, which supported the increase in aquaporin expression (Ding et al., 2016b). Abscisic acid had a positive effect on Lpr and aquaporin expression (Aroca et al., 2006; Mahdieh and Mostajeran, 2009; Parent et al., 2009). Parent et al. (2009) demonstrated that a higher aquaporin expression and Lpr was observed in the maize line producing more ABA than in the line producing less ABA.

### Drought Stress Affects Lpr Through the Regulation of Root Anatomy and Morphology

The decrease in Lpr could be explained by increased or accelerated deposition of root suberin under drought stress (Gambetta et al., 2017), and the accumulation of suberin leads to the formation of apoplastic barriers. Vandeleur et al. (2009) demonstrated that the diminution of Lpr was caused by suberin and lignin depositions, which restricts the apoplastic water flow under drought stress. In rice plants, suberization of the endodermis increased under drought stress (Henry et al., 2012). On the other hand, more aerenchyma formation could restrict the passage of water through cortical cells in rice roots (Ranathunge et al., 2003, 2004; Yang et al., 2012; Ren et al., 2015). Yang et al. (2012) observed that drought induced more root aerenchyma formation and restricted root water uptake in rice plants supplied with nitrate.

Additionally, Lpr is regulated by the change in root morphology under drought stress. Plants tend to develop a deeper root system to obtain more water, since the drying rate is more pronounced in superficial soil layers than in the deeper ones (Pinheiro et al., 2005; Alsina et al., 2010). In rice plants, lateral root growth was enhanced by water stress treatment with PEG 6000 in plants supplied with ammonium (Ding et al., 2015).

## Drought Stress Affects Photosynthesis

Drought stress decreases photosynthetic rate (A), restricts plant growth, and reduces crop yield (Farooq et al., 2009). The decrease in A is associated with stomatal closure (Flexas and Medrano, 2002; Flexas et al., 2006a) and metabolic impairment (Tezara et al., 1999; Tang et al., 2002). In most studies, the decrease in A was due to stomatal closure and increase in resistance to CO_2_ diffusion (Xu et al., 2010; Flexas et al., 2012; Perez-Martin et al., 2014). Under drought stress, ABA accumulated in leaf apoplast and induced stomatal closure (Seki et al., 2007; Skirycz and Inzé, 2010; Rodrigues et al., 2017). Photosynthesis was restored after elevating CO_2_ concentration in leaves (Kaiser, 1987; Gallé et al., 2007) or stripping the epidermis (Schwab et al., 1989), indicating that stomatal closure is the main factor causing the decline in A. The ways to evaluate photosynthesis limitation under drought stress are discussed by Flexas et al. (2012). Drought stress intensity was divided into three levels based on stomatal conductance (g_s_): (1) mild drought stress (g_s_ > 0.15 mol H_2_O m^-2^ s^-1^), (2) moderate drought stress (0.05 mol H_2_O m^-2^ s^-1^< g_s_ < 0.15 mol H_2_O m^-2^ s^-1^), and (3) severe drought stress (g_s_ < 0.05 mol H_2_O m^-2^ s^-1^) (Medrano et al., 2002; Cano et al., 2014). During mild drought stress, decrease in g_s_ was the only cause for the decline in photosynthetic rate. During moderate drought stress, the decrease in g_s_ and meshophyll conductance (g_m_) caused the decline in A. After severe drought stress photosynthetic capacity is impaired, inhibiting photosynthetic enzymes and decreasing chlorophyll and protein content. The plants also suffer oxidative stress under severe drought stress (Zhou et al., 2007; Farooq et al., 2009). However, the decrease in g_s_ and g_m_ accounts for more than 90% of total A reduction from mild to severe drought stress in tobacco (Galle et al., 2009) and eucalyptus (Cano et al., 2014).

In C_3_ plants, light-saturated photosynthetic rate is restricted by chloroplastic CO_2_ concentration (Cc) under present ambient CO_2_ level, and Cc is unsaturated (Li et al., 2009b; Ding et al., 2016a). The Cc depends on the regulation of g_s_ and g_m_ (Flexas et al., 2008; Evans et al., 2009; Kaldenhoff, 2012). Under drought stress, even less Cc is predicted owing to stomatal closure, the increase in diffusion resistance, and the activity of Rubisco (key enzyme for carboxylation), which decreases due to insufficient CO_2_ (Flexas et al., 2006a). In comparison with stomatal closure, which is regulated by ABA and/or hydrogen peroxide (H_2_O_2_) (Zhang et al., 2001; Rodrigues et al., 2017), the regulation of g_m_ is more complex under drought stress. It was demonstrated that the decrease in Ψ_leaf_ resulted in chloroplast downsizing and subsequently decreased g_m_ in plants supplied with nitrate under water stress treatment with PEG 6000 (Li et al., 2012). Chloroplast shrinking induced the decrease in total chloroplast surface area and the surface area of chloroplasts exposed to intercellular airspace per unit leaf area (Sc), which are positively correlated to g_m_ (Evans et al., 2009; Li et al., 2009b; Xiong et al., 2017).

In other studies, the decrease in g_s_ and g_m_ has been associated with the regulation of aquaporin expression (Flexas et al., 2006b; Miyazawa et al., 2008; Pou et al., 2013; Perez-Martin et al., 2014). In olive, the downregulation of two aquaporin gene expression, *OePIP1;1* and *OePIP2;1*, explained the decrease in both g_s_ and g_m_ under drought stress (Perez-Martin et al., 2014). Pou et al. (2013) observed that the expression of *VvTIP2;1*, a grapevine tonoplast aquaporin, was highly correlated with g_s_, and the downregulated expression might partially cause g_s_ decline under drought stress. However, they also found that there was no decrease in the expression of the other aquaporin genes under drought stress, for example, *VvPIP2;1* (a grapevine root-specific aquaporin) and *VvTIP1;1* (an isoform of the grapevine tonoplast aquaporin). This result suggests that the aquaporin members play different roles in regulating leaf water relations and photosynthesis. Indeed, some aquaporin genes are located in stomatal complexes [guard cells, Hachez et al. (2017)], and they are involved in controlling the stomatal movement. Rodrigues et al. (2017) showed that AtPIP2;1, an aquaporin in Arabidopsis, facilitated H_2_O_2_ entry into guard cells and induced stomatal closure under ABA treatment. Evidence elucidates that the inhibition of aquaporin expression in bundle sheath cells was due to ABA accumulation in leaf under drought stress (Shatil-Cohen et al., 2011). Mizokami et al. (2015) observed that g_m_ decreased with the increase in leaf ABA content in wild type plants under drought stress, whereas both ABA and g_m_ were unchanged in *aba*1, an ABA-deficient mutant, indicating that ABA plays a major role in the regulation of g_m_ under drought stress by affecting aquaporin expression.

Full recovery of A after rewatering was observed in many studies (Izanloo et al., 2008; Xu et al., 2009; Cano et al., 2014). However, the recovery speed varied among these studies, which depended on the degree and velocity of decline in A during stress imposition (Flexas et al., 2012). In severe drought stress plants, the recovery of A was only 40–60% on the first day of rewatering, but the recovery continued in the next few days. When A was 36% in control plants before rewatering, the total recovery of A occurred in 4 days. When A was 23% in control plants, full recovery took up to 6 days, and when A was 3% in control plants, full recovery required 18 days (Flexas et al., 2012). Besides, the recovery of A depends on the change in g_s_ and g_m_ after rewatering. Cano et al. (2014) observed that full recovery of A was associated with quick recovery of g_m_ in eucalyptus, whereas g_s_ recovery was slower than g_m_. Stomatal conductance might not be fully recovered after rewatering, which aims to increase intrinsic water use efficiency (Gallé et al., 2007; Galmés et al., 2007; Xu et al., 2009).

## The Coordinated Decline in K_leaf_ With a Under Drought Stress

The coordination between K_leaf_ and A played an important role in the evolution of leaves (Sack and Holbrook, 2006; Scoffoni et al., 2016). Many studies have demonstrated that positive correlations exist among species between hydraulic conductance of stem; leaf; the whole plant; and g_s_, g_m_, and A (Sack and Holbrook, 2006; Brodribb et al., 2007; Flexas et al., 2013; Scoffoni et al., 2016; Xiong et al., 2017).

Under drought stress, coordinated decline of K_leaf_ and A was observed in maize (Gleason et al., 2017), rice (Tabassum et al., 2016), and woodland species (Skelton et al., 2017). In rice plants, the decrease in major venation thickness induced the decline of both A and K_leaf_ (Tabassum et al., 2016). In other studies, it has been shown that ABA plays an important role in the coordinated decline of K_leaf_ and A under drought stress (Shatil-Cohen et al., 2011; Mizokami et al., 2015; Coupel-Ledru et al., 2017), through the regulation of aquaporins (Shatil-Cohen et al., 2011; Pou et al., 2013). Abscisic acid induced the deactivation of aquaporins in bundle sheath cells under drought stress, which caused the decrease in Ψ_leaf_ and K_leaf_ (Shatil-Cohen et al., 2011). The deactivation of aquaporins could directly downregulate g_m_ by affecting CO_2_ transport (Flexas et al., 2006b; Evans et al., 2009; Kaldenhoff, 2012).

## Cross Talk of N, Water Transport, and Drought Stress

### Nitrogen Supply Affects Root Water Uptake

Nitrogen is an essential macronutrient for plants, and it affects many aspects of plant growth and metabolic pathways (Guo et al., 2007c; Xu et al., 2012; Wang et al., 2014). Ammonium and nitrate are two major sources of N uptake by higher plants. The N form and the levels of N available affect root water uptake (Tyerman et al., 2017). Gorska et al. (2008a) found that the increase in root water uptake was associated with high nitrate supply (5 mM) in cucumber and tomato. Further analysis demonstrated that the increase in root hydraulic conductivity resulted from the change in Lpc, which was measured with a cell pressure probe. The Lpc decreased after inhibition of nitrate uptake by cucumber roots with nitrate reductase inhibitor tungstate, whereas Lpc was able to recover after direct injection of nitrate into the cells (Gorska et al., 2008a). Additionally, it was demonstrated that the capacity for nitrate regulation of Lpr correlated with the species’ nitrate uptake rates (Górska et al., 2010). High nitrate supply significantly increased the nitrate uptake rate, as well as root water uptake rate in maize plants, whereas the increase was not found in *Populus trichocarpa*, which is insensitive to high nitrate supply. Similar result was obtained by Li G. et al. (2016), although they showed a strong positive relationship between Lpr and nitrate accumulation in shoots rather than in roots. In *NRT2.1*, mutant of a high-affinity nitrate transporter, there was 30% reduction in Lpr. The results revealed that synergetic transport exists between nitrate and water uptake in roots. In plants supplied with N in both ammonium and nitrate forms, the high N supply also increased Lpr in rice (Ishikawa-Sakurai et al., 2014; Ren et al., 2015). Nitrogen deprivation decreased Lpr, resulting from the downregulation of aquaporin genes in roots, as well as the increased aerenchyma formation. On the contrary, high ammonium (3 mM) supply induced more apoplastic barrier formation and decreased Lpr when compared with low ammonium supply (0.03 mM) in rice seedlings (Ranathunge et al., 2016). Nonetheless, when we compared root water uptake in plants supplied with ammonium or nitrate, a higher expression of aquaporin genes (*PIPs* and *TIPs*) was observed in rice plants supplied with ammonium than with nitrate (2.86 mM) (Ding et al., 2016b; Wang et al., 2016), indicating a higher water uptake ability (symplastic pathway flux) in rice plants under similar conditions. But, this was not observed in other species, such as maize (Gorska et al., 2008b) and French bean (Guo et al., 2007b). Instead, they observed higher root water uptake or aquaporin expression in plants supplied with nitrate than with ammonium.

With different forms of N supply, the regulation of root hydraulics/aquaporins could be through (1) local and systemic signaling induced by nitrate (Cramer et al., 2009; Li G. et al., 2016), (2) root anatomy development, i.e., the depositions of lignin and suberin, regulated by ammonium and nitrate (Ren et al., 2015; Barberon et al., 2016; Ranathunge et al., 2016; Gao et al., 2017), or (3) the transport of N-containing molecules (Wang et al., 2016). Firstly, there is a strong correlation between soil N mobility and water mass flow. More nitrate could reach the root surface with increasing total water flow through the plant when nitrate is sensed (Gorska et al., 2008a,b; Cramer et al., 2009). Both high and low affinity nitrate transporters were involved in this sensing and signaling (Tyerman et al., 2017). In *NRT2.1* knock out plants, Lpr was reduced and under different N concentration treatments, Lpr was positively correlated with the nitrate content in leaves (Li G. et al., 2016). However, when the nitrate concentration was above 2 mM inside xylem, stomatal conductance decreased in an ABA-dependent manner in maize (Wilkinson et al., 2007). It could be expected that less water and nitrate were acquired. Secondly, it’s well known that two parallel pathways, namely, apoplastic and cell-to-cell pathway, exist for radial water movement in root. Basically, water flow in apoplastic pathway is blocked by apoplastic barriers, and water flow continues through the cell-to-cell pathway. The deposition of lignin and suberin may affect Lpr and the expression of aquaporins. Ranathunge et al. (2016) demonstrated that high ammonium supply increased the deposition of lignin and suberin; furthermore, Lpr decreased in comparison with low ammonium supply in rice. Unfortunately, they didn’t examine the difference between ammonium and nitrate supply. In a previous study, we observed that the expression of *PIPs* and *TIPs* was higher in rice plants supplied with ammonium than with nitrate (Ding et al., 2016b). We could expect a higher deposition of lignin and/suberin in roots supplied with ammonium than with nitrate, since no difference in Lpr was observed between ammonium and nitrate treatments (Yang et al., 2012; Ding et al., 2015). Moreover, the production of ethylene and ABA was regulated by the different N forms available in rice (Ding et al., 2015, 2016b; Gao et al., 2017). Ethylene may reduce the suberisation, whereas ABA increases the suberisation (Barberon et al., 2016). Thirdly, some aquaporin genes are involved in NH_4_^+^/NH_3_ transport but not in nitrate transport in plants (Wang et al., 2016). The correlation between nitrogen fixation and aquaporins is discussed in the next section. From this correlation, it becomes clear that the expression of aquaporins is regulated by ammonium/nitrate supply. Aquaporins could be regulated at many levels, including transcription, protein amount, localization, and by gating (Chaumont and Tyerman, 2014), and it remains unclear how N supply affects these regulations.

### The Correlation Between N Metabolism and Aquaporins

Nitrogen is acquired by plants through either nitrogen fixation from atmosphere, carried out by the Leguminosae family plants, or by utilization of N sources present in soil, including ammonium, nitrate, urea, and other organic N forms. During N absorption, assimilation, and remobilization, aquaporins play important roles, and the two main subfamilies involved are nodulin 26-like intrinsic proteins (NIPs) and tonoplast intrinsic proteins (TIPs).

#### Nodulin 26-Like Intrinsic Protein (NIPs) and Nitrogen Fixation

Nitrogen is fixed by Leguminosae family plants, through nodulin. Symbiosomes are established between nitrogen fixing bacteria and root by exchange of carbon and nitrogen through symbiosome membrane in the nodulin (Roth and Stacey, 1989; Mylona et al., 1995; Udvardi and Poole, 2013). Nodulin 26-like intrinsic protein is a superfamily of aquaporins (aquaglyceroporin), and it was named based on nodulin 26, which is the major protein component of the mature soybean symbiosome membrane (Fortin et al., 1987; Weaver et al., 1991; Kaldenhoff and Fischer, 2006). It was observed that, nodulin 26 was able to facilitate the transport of water and glycerol (Rivers et al., 1997; Dean et al., 1999) and the efflux of NH_3_/NH_4_^+^ from the symbiosome membrane based on stopped flow measurement with symbiosome membrane vesicles (Niemietz and Tyerman, 2000) and proteoliposomes by reconstituting nodulin 26 protein (Hwang et al., 2010). Nodulin 26 showed a fivefold preference in the transport rate of ammonia when compared with water (Hwang et al., 2010). Moreover, Masalkar et al. (2010) observed that nodulin 26 formed a complex with soybean nodule cytosolic glutamine synthetase (GS), which catalyzes the assimilation of ammonia. GS interacts with the carboxyl terminal domain of nodulin 26, by regulating the activity, trafficking, and stability of nodulin 26. The results suggested that nodulin 26 plays a major role in nitrogen fixation by Leguminosae plants. Phosphorylation of nodulin 26 was induced by osmotic drought stress (Guenther et al., 2003) and flooding/hypoxia stress (Hwang, 2013), by affecting the activity of water and/or ammonia transport.

Additionally, the expression of NIPs was induced by arbuscular mycorrhizal (AM) fungi infection in *Lotus japonicas* (Giovannetti et al., 2012) and *Medicago truncatula* (Uehlein et al., 2007), which benefits the utilization of phosphate and nitrogen (Smith and Smith, 2011). It could be assumed that NIPs are involved in both rhizobial and AM symbiosis for nutrient delivery and water transport.

During the evolution of plants, NIPs were present in all land plants (Roberts and Routray, 2017), such as maize (Chaumont et al., 2001), Arabidopsis (Johanson et al., 2001), rice (Sakurai et al., 2005), grapevine (Fouquet et al., 2008), cotton (Park et al., 2010), and soybean (Zhang et al., 2013). Apart from their function as ammonia channels, NIPs are also characterized as channels for metalloids (Bienert and Bienert, 2017), including boron (Takano et al., 2008), silicon (Ma and Yamaji, 2015), arsenic (Ma et al., 2008), aluminum (Wang Y. et al., 2017), antimony, and germanium (Bienert and Bienert, 2017). For more details, the function and classification of NIPs were reviewed by Roberts and Routray (2017).

#### Aquaporin Facilitates the Transport of Ammonium, Ammonia, and Urea

Urea is the most widely used nitrogen fertilizer in agricultural crop production and also an important N metabolite in plants. Urea is degraded to ammonium by urease in soil and then utilized by plants. However, urea can be taken up by roots directly, mediated by two types transporters, namely, aquaporins (Liu et al., 2003b; Yang et al., 2015) and DUR3 orthologs (Liu et al., 2003a; Witte, 2011). Wang et al. (2016), in a review, showed that two main subfamilies of aquaporins were involved in the transport of urea, including NIPs and TIPs. Nodulin 26-like proteins facilitate the entry of urea into cells via the plasma membrane, followed by vacuolar loading through TIPs. Vacuolar loading is beneficial for the storage of excess urea, and vacuolar unloading can remobilize the urea under nitrogen starvation (Kojima et al., 2006). Zhang et al. (2016) demonstrated that CsNIP2;1, a plasma membrane transporter from *Cucumis sativus*, was able to transport urea through the plasma membrane when expressed in yeast. The expression of *CsNIP2;1* was induced by nitrogen deficiency. Additionally, they found that ectopic expression of *CsNIP2;1* improved the growth of Arabidopsis and rescued the growth of *atdur3-3* mutant on medium with urea as the sole N source. These results suggested that urea was transported by aquaporins of NIPs, which were localized in the plasma membrane. On the contrary, a lower expression of *AtNIP5;1* and *AtNIP6;1*, two urea transporters, was observed in Arabidopsis supplied with urea than with ammonium nitrate, although a higher expression of DUR3 was observed in the plants under similar conditions (Yang et al., 2015). It was postulated that the downregulation of *AtNIP5;1* and *AtNIP6;1* was involved in the detoxicification of urea/ammonia under excessive urea level. Besides, it was demonstrated that urea uptake decreased in *nip5;1* when compared with the wild type under boron deficient conditions. The remobilization of urea from vacuoles is regulated by TIPs. ZmTIP4;4, a maize aquaporin gene, was shown to facilitate the transport of urea, and the expression of the gene was upregulated under N deficiency in expanded leaves (Gu et al., 2012), suggesting that ZmTIP4;4 played an important role in unloading vacuolar urea across tonoplast under N deficient conditions. Soto et al. (2010) demonstrated that two urea transporters were involved in N recycling in pollen tubes in Arabidopsis.

Urea is degraded to ammonium by the enzyme urease present in soil. Ammonia (NH_4_^+^/NH_3_) is taken up by roots mainly through ammonium transporters (Xu et al., 2012). Transport of NH_4_^+^/NH_3_ into vacuole would allow N storage and eliminate toxicity, and the stored N could be remobilized by passive and low-affinity transport pathways. Both the influx and efflux of NH_4_^+^/NH_3_ into vacuole are regulated by TIPs (Wang et al., 2016).

### Nitrogen Supply Affects Drought Tolerance in Plants

Despite the high nitrate supply, increased root water uptake was observed under normal water condition, and the high nitrate supply may decrease drought tolerance in plants under drought stress. Wilkinson et al. (2007) observed that the decrease in stomatal closure and leaf elongation rates were more sensitive to drought stress in maize plants supplied with high nitrate. Stomatal conductance decreased by 30% in plants supplied with high nitrate after 3 days of drought stress, whereas only 10% decrease in g_s_ was found in control plants (supplied with water). Further evidence showed that the effect of nitrate on growth inhibition under drought stress was associated with pH based ABA redistribution. Drought stress may induce the alkalinization of leaf apoplast, in tomato (Jia and Davies, 2007) and hop (Korovetska et al., 2014), and especially in plants supplied with high nitrate (Wilkinson et al., 2007). While pH increases under drought stress, more ABA is activated in leaf apoplast, which further induces stomatal closure (Zhang et al., 2006) and decrease in K_leaf_ (Shatil-Cohen et al., 2011).

On the other hand, nitrogen supply might affect plant drought tolerance through regulation of root water uptake (**Figure 1**). In plants supplied with ammonium nutrition, drought stress induced a rapid decrease in aquaporin expression (including PIPs and TIPs), meanwhile ABA started to accumulate in the roots (Ding et al., 2016b). After 24 h water stress treatment with PEG 6000, an increase in aquaporin expression was observed, and ABA accumulation reached a peak. Both increase in aquaporin expression and Lpr were regulated by ABA accumulation (Ding et al., 2015, 2016b). In plants supplied with nitrate, root water uptake and transport were restricted by lower aquaporin expression and/or activity, more aerenchyma formation was observed when compared with plants supplied with ammonium under water stress treatment with PEG 6000. Yang et al. (2012) investigated that more aerenchyma formation would restrict radial water transport in roots supplied with nitrate than with ammonium, and aerenchyma formation was regulated by ethylene production (Gao et al., 2017). Additionally, ethylene may inhibit ABA production (Sharp, 2002), which could further affect aquaporin expression.

Interestingly, increased root ABA content and higher stomatal conductance were found in rice plants supplied with ammonium than with nitrate under water stress treatment with PEG 6000 (Ding et al., 2016b). It’s well known that drought stress induces stomatal closure, regulated by ABA; yet, this ABA may be not from roots. Christmann et al. (2007) showed that this ABA was biosynthesized in shoots and it further induced stomatal closure.

## Implications

Many efforts have been made to increase crop drought resistance through identification of genetic, transcriptomic, metabolomic, and epigenetic aspects. Water uptake and photosynthesis are the two key traits that enhance crop drought tolerance. In this review, two approaches have been highlighted for enhancing crop drought tolerance:

(1) Deregulation of aquaporin expression. Many researchers have demonstrated that over-expressing a single aquaporin gene could enhance plant drought tolerance and silence the genes that result in drought sensitivity in plants (**Table 1**). There are plenty of aquaporin members in plant species (Fox et al., 2017), and they play important roles in controlling water relations (Chaumont and Tyerman, 2014), nutrient uptake (Wang et al., 2016), and photosynthesis (Groszmann et al., 2017; Uehlein et al., 2017). In the future, more aquaporin genes should be characterized and their expression should be genetically modified in specific tissues and/or organs to enhance plant drought tolerance.
(2) Ammonium fertilizer application for rice water saving culture. Rice is traditionally cultivated in waterlogged conditions, and 80% of the freshwater used in agriculture is for rice production in China (Guo et al., 2007a). With increase in the severity of water shortage, water saving culture (non-flooded mulching cultivation) has become popular now. The main nutritional change that occurs when rice is cultivated in aerobic soil is the N form, i.e., from ammonium in waterlogged condition, to nitrate and/or the mixture of ammonium and nitrate in aerobic condition. It was well documented that ammonium nutrition could enhance rice seedling drought tolerance (Guo et al., 2007a; Li et al., 2009a). In non-flooded mulching cultivation of rice, we recommend using ammonium fertilizer to enhance drought tolerance in rice seedlings.

**Table 1:** Drought tolerance was affected by the deregulation of a single aquaporin gene.

Deregulation	Drought tolerance	Species	Genes	Reference
Over-expression	Drought tolerant	Arabidopsis	JcPIP2;7/JcTIP1;3	Khan et al., 2015
		Arabidopsis	AvNIP5;1	Yu et al., 2015
		Arabidopsis	FaPIP2;1	Zhuang et al., 2015
		Arabidopsis	MaPIP1;1	Xu et al., 2014
		Arabidopsis	PgTIP1	Peng et al., 2007
		Tobacco	BjPIP1	Zhang et al., 2008
		Tobacco	BnPIP1	Yu et al., 2005
		Banana	MusaPIP1;2	Sreedharan et al., 2013
		Tomato	MdPIP1;3	Wang L. et al., 2017
		Tomato	SlPIP2;1/SlPIP2;5/SlPIP2;7	Li R. et al., 2016
		Tomato	SlTIP2;2	Sade et al., 2009
		Rice	RWC3	Lian et al., 2004
		Soybean	GmTIP2;1	Zhang et al., 2017
Down-regulation	Drought sensitive	Arabidopsis	PIP1/PIP2	Martre et al., 2002
		Tobacco	NtAQP1	Siefritz et al., 2002
		Tobacco	BnPIP1	Yu et al., 2005
		Poplar	PIP1	Secchi and Zwieniecki, 2014


## Author Contributions

LD and SG wrote the manuscript. ZL, LG, and QS contributed to the discussion and revision of the manuscript.

## Conflict of Interest Statement

The authors declare that the research was conducted in the absence of any commercial or financial relationships that could be construed as a potential conflict of interest.

## References

[B1] Alsina M. M. Smart D. R. Bauerle T. De Herralde F. Biel C. Stockert C. (2010). Seasonal changes of whole root system conductance by a drought-tolerant grape root system. *J. Exp. Bot.* 62 99–109. 10.1093/jxb/erq247 20851906 PMC2993904

[B2] Aroca R. Ferrante A. Vernieri P. Chrispeels M. J. (2006). Drought, abscisic acid and transpiration rate effects on the regulation of PIP aquaporin gene expression and abundance in *Phaseolus vulgaris* plants. *Ann. Bot.* 98 1301–1310. 10.1093/aob/mcl219 17028296 PMC2803586

[B3] Aroca R. Porcel R. Ruiz-Lozano J. M. (2012). Regulation of root water uptake under abiotic stress conditions. *J. Exp. Bot.* 63 43–57. 10.1093/jxb/err266 21914658

[B4] Aroca R. Ruiz-Lozano J. M. (2012). “Regulation of root water uptake under drought stress conditions,” in *Plant Responses to Drought Stress*, ed. Aroca R. (Berlin: Springer), 113–127.

[B5] Barberon M. Vermeer J. E. M. De Bellis D. Wang P. Naseer S. Andersen T. G. (2016). Adaptation of root function by nutrient-induced plasticity of endodermal differentiation. *Cell* 164 447–459. 10.1016/j.cell.2015.12.021 26777403

[B6] Bienert M. D. Bienert G. P. (2017). “Plant aquaporins and metalloids,” in *Plant Aquaporins*, Chaumont F. Tyerman S. D. (Cham: Springer), 297–332.

[B7] Brodribb T. J. Feild T. S. Jordan G. J. (2007). Leaf maximum photosynthetic rate and venation are linked by hydraulics. *Plant Physiol.* 144 1890–1898. 10.1104/pp.107.101352 17556506 PMC1949879

[B8] Buckley T. N. (2015). The contributions of apoplastic, symplastic and gas phase pathways for water transport outside the bundle sheath in leaves. *Plant Cell Environ.* 38 7–22. 10.1111/pce.12372 24836699

[B9] Cano F. J. López R. Warren C. R. (2014). Implications of the mesophyll conductance to CO2 for photosynthesis and water-use efficiency during long-term water stress and recovery in two contrasting *Eucalyptus* species. *Plant Cell Environ.* 37 2470–2490. 10.1111/pce.12325 24635724

[B10] Chang C. (2007). “Impacts, Adaptation and Vulnerability,” in *Proceedings of the Contribution of Working Group II to the Fourth Assessment Report of the Intergovernmental Panel on Climate Change/ML Parry*, eds Canziani O. F. Palutikof J. P., (Cambridge: Cambridge University Press), 976.

[B11] Chaumont F. Barrieu F. Wojcik E. Chrispeels M. J. Jung R. (2001). Aquaporins constitute a large and highly divergent protein family in maize. *Plant Physiol.* 125 1206–1215. 10.1104/pp.125.3.1206 11244102 PMC65601

[B12] Chaumont F. Tyerman S. D. (2014). Aquaporins: highly regulated channels controlling plant water relations. *Plant Physiol.* 164 1600–1618. 10.1104/pp.113.233791 24449709 PMC3982727

[B13] Chaves M. M. Maroco J. P. Pereira J. S. (2003). Understanding plant responses to drought—from genes to the whole plant. *Funct. Plant Biol.* 30 239–264. 10.1071/FP02076 32689007

[B14] Christmann A. Weiler E. W. Steudle E. Grill E. (2007). A hydraulic signal in root-to-shoot signalling of water shortage. *Plant J.* 52 167–174. 10.1111/j.1365-313X.2007.03234.x 17711416

[B15] Coupel-Ledru A. Tyerman S. Masclef D. Lebon E. Christophe A. Edwards E. J. (2017). Abscisic acid down-regulates hydraulic conductance of grapevine leaves in isohydric genotypes only. *Plant Physiol.* 175 1121–1134. 10.1104/pp.17.00698 28899961 PMC5664463

[B16] Cramer M. D. Hawkins H.-J. Verboom G. A. (2009). The importance of nutritional regulation of plant water flux. *Oecologia* 161 15–24. 10.1007/s00442-009-1364-3 19449035

[B17] Dean R. M. Rivers R. L. Zeidel M. L. Roberts D. M. (1999). Purification and functional reconstitution of soybean nodulin 26. An aquaporin with water and glycerol transport properties. *Biochemistry* 38 347–353. 10.1021/bi982110c 9890916

[B18] Ding L. Gao C. Li Y. Li Y. Zhu Y. Xu G. (2015). The enhanced drought tolerance of rice plants under ammonium is related to aquaporin (AQP). *Plant Sci.* 234 14–21. 10.1016/j.plantsci.2015.01.016 25804805

[B19] Ding L. Gao L. Liu W. Wang M. Gu M. Ren B. (2016a). Aquaporin plays an important role in mediating chloroplastic CO2 concentration under high-N supply in rice (*Oryza sativa*) plants. *Physiol. Plant.* 156 215–226. 10.1111/ppl.12387 26382720

[B20] Ding L. Li Y. Wang Y. Gao L. Wang M. Chaumont F. (2016b). Root ABA accumulation enhances rice seedling drought tolerance under ammonium supply: interaction with aquaporins. *Front. Plant Sci.* 7:1206. 10.3389/fpls.2016.01206 27559341 PMC4979525

[B21] Evans J. R. Kaldenhoff R. Genty B. Terashima I. (2009). Resistances along the CO2 diffusion pathway inside leaves. *J. Exp. Bot.* 60 2235–2248. 10.1093/jxb/erp117 19395390

[B22] Farooq M. Wahid A. Kobayashi N. Fujita D. Basra S. (2009). *Plant Drought Stress: Effects, Mechanisms and Management Sustainable Agriculture.* Berlin: Springer), 153–188.

[B23] Flexas J. Gallé A. Galmés J. Ribas-Carbo M. Medrano H. (2012). “The response of photosynthesis to soil water stress,” in *Plant Responses to Drought Stress*, ed. Aroca R. (Berlin: Springer), 129–144. 10.1007/978-3-642-32653-0_5

[B24] Flexas J. Medrano H. (2002). Drought-inhibition of photosynthesis in C3 plants: stomatal and non-stomatal limitations revisited. *Ann. Bot.* 89 183–189. 10.1093/aob/mcf027 12099349 PMC4233792

[B25] Flexas J. Ribas-Carbo M. Diaz-Espejo A. Galmes J. Medrano H. (2008). Mesophyll conductance to CO2: current knowledge and future prospects. *Plant Cell Environ.* 31 602–621. 10.1111/j.1365-3040.2007.01757.x 17996013

[B26] Flexas J. Ribas-Carbó M. Bota J. Galmés J. Henkle M. Martínez-Cañellas S. (2006a). Decreased Rubisco activity during water stress is not induced by decreased relative water content but related to conditions of low stomatal conductance and chloroplast CO2 concentration. *New Phytol.* 172 73–82. 10.1111/j.1469-8137.2006.01794.x 16945090

[B27] Flexas J. Ribas-Carbo M. Hanson D. T. Bota J. Otto B. Cifre J. (2006b). Tobacco aquaporin NtAQP1 is involved in mesophyll conductance to CO2 *in vivo*. *Plant J.* 48 427–439. 10.1111/j.1365-313X.2006.02879.x 17010114

[B28] Flexas J. Scoffoni C. Gago J. Sack L. (2013). Leaf mesophyll conductance and leaf hydraulic conductance: an introduction to their measurement and coordination. *J. Exp. Bot.* 64 3965–3981. 10.1093/jxb/ert319 24123453

[B29] Fortin M. G. Morrison N. A. Verma D. P. S. (1987). Nodulin-26, a peribacteroid membrane nodulin is expressed independently of the development of the peribacteroid compartment. *Nucleic Acids Res.* 15 813–824. 10.1093/nar/15.2.813 3822816 PMC340469

[B30] Fouquet R. Léon C. Ollat N. Barrieu F. (2008). Identification of grapevine aquaporins and expression analysis in developing berries. *Plant Cell Rep.* 27 1541–1550. 10.1007/s00299-008-0566-1 18560835

[B31] Fox A. R. Maistriaux L. C. Chaumont F. (2017). Toward understanding of the high number of plant aquaporin isoforms and multiple regulation mechanisms. *Plant Sci.* 264 179–187. 10.1016/j.plantsci.2017.07.021 28969798

[B32] Galle A. Florez-Sarasa I. Tomas M. Pou A. Medrano H. Ribas-Carbo M. (2009). The role of mesophyll conductance during water stress and recovery in tobacco (*Nicotiana sylvestris*): acclimation or limitation? *J. Exp. Bot.* 60 2379–2390. 10.1093/jxb/erp071 19321646

[B33] Gallé A. Haldimann P. Feller U. (2007). Photosynthetic performance and water relations in young pubescent oak (*Quercus pubescens*) trees during drought stress and recovery. *New Phytol.* 174 799–810. 10.1111/j.1469-8137.2007.02047.x 17504463

[B34] Galmés J. Medrano H. Flexas J. (2007). Photosynthetic limitations in response to water stress and recovery in Mediterranean plants with different growth forms. *New Phytol.* 175 81–93. 10.1111/j.1469-8137.2007.02087.x 17547669

[B35] Gambetta G. A. Knipfer T. Fricke W. Mcelrone A. J. (2017). “Aquaporins and Root Water Uptake,” in *Plant Aquaporins: From Transport to Signaling*, eds Chaumont F. Tyerman S. D.(Cham: Springer), 133–153. 10.1007/978-3-319-49395-4_6

[B36] Gao C. Ding L. Li Y. Chen Y. Zhu J. Gu M. (2017). Nitrate increases ethylene production and aerenchyma formation in roots of lowland rice plants under water stress. *Funct. Plant Biol.* 44 430–442. 10.1071/FP16258 32480576

[B37] Gao Y. X. Li Y. Yang X. X. Li H. J. Shen Q. R. Guo S. W. (2010). Ammonium nutrition increases water absorption in rice seedlings (*Oryza sativa* L.) under water stress. *Plant Soil* 331 193–201. 10.1007/s11104-009-0245-1

[B38] Geber M. A. Dawson T. E. (1990). Genetic variation in and covariation between leaf gas exchange, morphology, and development in *Polygonum arenastrum*, an annual plant. *Oecologia* 85 153–158. 10.1007/BF00319396 28312550

[B39] Giovannetti M. Balestrini R. Volpe V. Guether M. Straub D. Costa A. (2012). Two putative-aquaporin genes are differentially expressed during arbuscular mycorrhizal symbiosis in *Lotus japonicus*. *BMC Plant Biol.* 12:186. 10.1186/1471-2229-12-186 23046713 PMC3533510

[B40] Gleason S. M. Wiggans D. R. Bliss C. A. Comas L. H. Cooper M. Dejonge K. C. (2017). Coordinated decline in photosynthesis and hydraulic conductance during drought stress in *Zea mays*. *Flora* 227 1–9. 10.1016/j.flora.2016.11.017

[B41] Górska A. Lazor J. W. Zwieniecka A. K. Benway C. Zwieniecki M. A. (2010). The capacity for nitrate regulation of root hydraulic properties correlates with species’ nitrate uptake rates. *Plant Soil* 337 447–455. 10.1007/s11104-010-0540-x

[B42] Gorska A. Ye Q. Holbrook N. M. Zwieniecki M. A. (2008a). Nitrate control of root hydraulic properties in plants: translating local information to whole plant response. *Plant Physiol.* 148 1159–1167. 10.1104/pp.108.122499 18753287 PMC2556825

[B43] Gorska A. Zwieniecka A. Holbrook N. M. Zwieniecki M. A. (2008b). Nitrate induction of root hydraulic conductivity in maize is not correlated with aquaporin expression. *Planta* 228 989–998. 10.1007/s00425-008-0798-x 18679712

[B44] Grondin A. Mauleon R. Vadez V. Henry A. (2016). Root aquaporins contribute to whole plant water fluxes under drought stress in rice (*Oryza sativa* L.). *Plant Cell Environ.* 39 347–365. 10.1111/pce.12616 26226878

[B45] Groszmann M. Osborn H. L. Evans J. R. (2017). Carbon dioxide and water transport through plant aquaporins. *Plant Cell Environ.* 40 938–961. 10.1111/pce.12844 27739588

[B46] Gu R. Chen X. Zhou Y. Yuan L. (2012). Isolation and characterization of three maize aquaporin genes, ZmNIP2; 1, ZmNIP2; 4 and ZmTIP4; 4 involved in urea transport. *BMB Rep.* 45 96–101. 10.5483/BMBRep.2012.45.2.96 22360887

[B47] Guenther J. F. Chanmanivone N. Galetovic M. P. Wallace I. S. Cobb J. A. Roberts D. M. (2003). Phosphorylation of soybean nodulin 26 on serine 262 enhances water permeability and is regulated developmentally and by osmotic signals. *Plant Cell* 15 981–991. 10.1105/tpc.009787 12671092 PMC152343

[B48] Guo S. Bruck H. Sattelmacher B. (2002). Effects of supplied nitrogen form on growth and water uptake of French bean (*Phaseolus vulgaris* L.) plants - Nitrogen form and water uptake. *Plant Soil* 239 267–275. 10.1023/A:1015014417018

[B49] Guo S. Chen G. Zhou Y. Shen Q. R. (2007a). Ammonium nutrition increases photosynthesis rate under water stress at early development stage of rice (*Oryza sativa* L.). *Plant Soil* 296 115–124. 10.1007/s11104-007-9302-9

[B50] Guo S. Kaldenhoff R. Uehlein N. Sattelmacher B. Brueck H. (2007b). Relationship between water and nitrogen uptake in nitrate- and ammonium-supplied *Phaseolus vulgaris* L. plants. *J. Plant Nutr. Soil Sci.* 170 73–80. 10.1002/jpln.200625073

[B51] Guo S. Zhou Y. Shen Q. Zhang F. (2007c). Effect of ammonium and nitrate nutrition on some physiological processes in higher plants - Growth, photosynthesis, photorespiration, and water relations. *Plant Biol.* 9 21–29. 10.1055/s-2006-924541 17048140

[B52] Hachez C. Milhiet T. Heinen R. B. Chaumont F. (2017). “Roles of Aquaporins in Stomata,” in *Plant Aquaporins*, eds Chaumont F. Tyerman S. (Cham: Springer), 167–183. 10.1007/978-3-319-49395-4_8

[B53] Hachez C. Veselov D. Ye Q. Reinhardt H. Knipfer T. Fricke W. (2012). Short-term control of maize cell and root water permeability through plasma membrane aquaporin isoforms. *Plant Cell Environ.* 35 185–198. 10.1111/j.1365-3040.2011.02429.x 21950760

[B54] Henry A. Cal A. J. Batoto T. C. Torres R. O. Serraj R. (2012). Root attributes affecting water uptake of rice (*Oryza sativa*) under drought. *J. Exp. Bot.* 63 4751–4763. 10.1093/jxb/ers150 22791828 PMC3427995

[B55] Hose E. Steudle E. Hartung W. (2000). Abscisic acid and hydraulic conductivity of maize roots: a study using cell-and root-pressure probes. *Planta* 211 874–882. 10.1007/s004250000412 11144273

[B56] Hwang J. H. (2013). *Soybean Nodulin 26: A Channel for Water and Ammonia at the Symbiotic Interface of Legumes and Nitrogen-Fixing Rhizobia Bacteria.* Ph.D. dissociation, Knoxville, TN, University of Tennessee.

[B57] Hwang J. H. Ellingson S. R. Roberts D. M. (2010). Ammonia permeability of the soybean nodulin 26 channel. *FEBS Lett.* 584 4339–4343. 10.1016/j.febslet.2010.09.033 20875821

[B58] Ishikawa-Sakurai J. Hayashi H. Murai-Hatano M. (2014). Nitrogen availability affects hydraulic conductivity of rice roots, possibly through changes in aquaporin gene expression. *Plant Soil* 379 289–300. 10.1007/s11104-014-2070-4

[B59] Iuchi S. Kobayashi M. Taji T. Naramoto M. Seki M. Kato T. (2001). Regulation of drought tolerance by gene manipulation of 9-*cis*-epoxycarotenoid dioxygenase, a key enzyme in abscisic acid biosynthesis in Arabidopsis. *Plant J.* 27 325–333. 10.1046/j.1365-313x.2001.01096.x 11532178

[B60] Izanloo A. Condon A. G. Langridge P. Tester M. Schnurbusch T. (2008). Different mechanisms of adaptation to cyclic water stress in two South Australian bread wheat cultivars. *J. Exp. Bot.* 59 3327–3346. 10.1093/jxb/ern199 18703496 PMC2529232

[B61] Jackson R. B. Sperry J. S. Dawson T. E. (2000). Root water uptake and transport: using physiological processes in global predictions. *Trends Plant Sci.* 5 482–488. 10.1016/S1360-1385(00)01766-0 11077257

[B62] Javot H. Maurel C. (2002). The role of aquaporins in root water uptake. *Ann. Bot.* 90 301–313. 10.1093/aob/mcf199 12234142 PMC4240399

[B63] Jia W. Davies W. J. (2007). Modification of leaf apoplastic pH in relation to stomatal sensitivity to root-sourced abscisic acid signals. *Plant Physiol.* 143 68–77. 10.1104/pp.106.089110 17098853 PMC1761989

[B64] Johanson U. Karlsson M. Johansson I. Gustavsson S. Sjövall S. Fraysse L. (2001). The complete set of genes encoding major intrinsic proteins in Arabidopsis provides a framework for a new nomenclature for major intrinsic proteins in plants. *Plant Physiol.* 126 1358–1369. 10.1104/pp.126.4.1358 11500536 PMC117137

[B65] Johnson D. M. Meinzer F. C. Woodruff D. R. Mcculloh K. A. (2009). Leaf xylem embolism, detected acoustically and by cryo-SEM, corresponds to decreases in leaf hydraulic conductance in four evergreen species. *Plant Cell Environ.* 32 828–836. 10.1111/j.1365-3040.2009.01961.x 19220781

[B66] Kaiser W. M. (1987). Effects of water deficit on photosynthetic capacity. *Physiol. Plant.* 71 142–149. 10.1111/j.1399-3054.1987.tb04631.x

[B67] Kaldenhoff R. (2012). Mechanisms underlying CO2 diffusion in leaves. *Curr. Opin. Plant Biol.* 15 276–281. 10.1016/j.pbi.2012.01.011 22300606

[B68] Kaldenhoff R. Fischer M. (2006). Functional aquaporin diversity in plants. *Biochim. Biophys. Acta* 1758 1134–1141. 10.1016/j.bbamem.2006.03.012 16730645

[B69] Khan K. Agarwal P. Shanware A. Sane V. A. (2015). Heterologous expression of two Jatropha aquaporins imparts drought and salt tolerance and improves seed viability in transgenic *Arabidopsis thaliana*. *PLoS One* 10:e0128866. 10.1371/journal.pone.0128866 26067295 PMC4466373

[B70] Kojima S. Bohner A. Von Wirén N. (2006). Molecular mechanisms of urea transport in plants. *J. Membr. Biol.* 212 83–91. 10.1007/s00232-006-0868-6 17264988

[B71] Korovetska H. Novák O. Jùza O. Gloser V. (2014). Signalling mechanisms involved in the response of two varieties of *Humulus lupulus* L. to soil drying: I. changes in xylem sap pH and the concentrations of abscisic acid and anions. *Plant Soil* 380 375–387. 10.1007/s11104-014-2101-1

[B72] Lambers H. Chapin F. III Pons T. (2008). *Plant Physiological Ecology*, 2nd Edn. New York, NY: Springer-Verlag 10.1007/978-0-387-78341-3

[B73] Li G. Tillard P. Gojon A. Maurel C. (2016). Dual regulation of root hydraulic conductivity and plasma membrane aquaporins by plant nitrate accumulation and high-affinity nitrate transporter NRT2. 1. *Plant Cell Physiol.* 57 733–742. 10.1093/pcp/pcw022 26823528

[B74] Li R. Wang J. Li S. Zhang L. Qi C. Weeda S. (2016). Plasma membrane intrinsic proteins *SlPIP2; 1, SlPIP2; 7* and *SlPIP2; 5* conferring enhanced drought stress tolerance in tomato. *Sci. Rep.* 6:31814. 10.1038/srep31814 27545827 PMC4992886

[B75] Li Y. Gao Y. X. Ding L. Shen Q. R. Guo S. W. (2009a). Ammonium enhances the tolerance of rice seedlings (*Oryza sativa* L.) to drought condition. *Agric. Water Manag.* 96 1746–1750. 10.1016/j.agwat.2009.07.008

[B76] Li Y. Gao Y. X. Xu X. M. Shen Q. R. Guo S. W. (2009b). Light-saturated photosynthetic rate in high-nitrogen rice (*Oryza sativa* L.) leaves is related to chloroplastic CO2 concentration. *J. Exp. Bot.* 60 2351–2360. 10.1093/jxb/erp127 19395387

[B77] Li Y. Ren B. B. Yang X. X. Xu G. H. Shen Q. R. Guo S. W. (2012). Chloroplast downsizing under nitrate nutrition restrained mesophyll conductance and photosynthesis in rice (*Oryza sativa* L.) under drought conditions. *Plant Cell Physiol.* 53 892–900. 10.1093/pcp/pcs032 22433461

[B78] Lian H.-L. Yu X. Ye Q. Ding X.-S. Kitagawa Y. Kwak S.-S. (2004). The role of aquaporin RWC3 in drought avoidance in rice. *Plant Cell Physiol.* 45 481–489. 10.1093/pcp/pch058 15111723

[B79] Liu L.-H. Ludewig U. Frommer W. B. Von Wirén N. (2003a). AtDUR3 encodes a new type of high-affinity urea/H+ symporter in Arabidopsis. *Plant Cell* 15 790–800. 10.1105/tpc.007120 12615950 PMC150031

[B80] Liu L.-H. Ludewig U. Gassert B. Frommer W. B. Von Wirén N. (2003b). Urea transport by nitrogen-regulated tonoplast intrinsic proteins in Arabidopsis. *Plant Physiol.* 133 1220–1228. 10.1104/pp.103.027409 14576283 PMC281617

[B81] Ma J. F. Yamaji N. (2015). A cooperative system of silicon transport in plants. *Trends Plant Sci.* 20 435–442. 10.1016/j.tplants.2015.04.007 25983205

[B82] Ma J. F. Yamaji N. Mitani N. Xu X.-Y. Su Y.-H. Mcgrath S. P. (2008). Transporters of arsenite in rice and their role in arsenic accumulation in rice grain. *Proc. Natl. Acad. Sci. U.S.A.* 105 9931–9935. 10.1073/pnas.0802361105 18626020 PMC2481375

[B83] Mahdieh M. Mostajeran A. (2009). Abscisic acid regulates root hydraulic conductance via aquaporin expression modulation in *Nicotiana tabacum*. *J. Plant Physiol.* 166 1993–2003. 10.1016/j.jplph.2009.06.001 19576659

[B84] Mahdieh M. Mostajeran A. Horie T. Katsuhara M. (2008). Drought stress alters water relations and expression of PIP-type aquaporin genes in *Nicotiana tabacum* plants. *Plant Cell Physiol.* 49 801–813. 10.1093/pcp/pcn054 18385163

[B85] Martre P. Morillon R. Barrieu F. North G. B. Nobel P. S. Chrispeels M. J. (2002). Plasma membrane aquaporins play a significant role during recovery from water deficit. *Plant Physiol.* 130 2101–2110. 10.1104/pp.009019 12481094 PMC166722

[B86] Masalkar P. Wallace I. S. Hwang J. H. Roberts D. M. (2010). Interaction of cytosolic glutamine synthetase of soybean root nodules with the C-terminal domain of the symbiosome membrane nodulin 26 aquaglyceroporin. *J. Biol. Chem.* 285 23880–23888. 10.1074/jbc.M110.135657 20504761 PMC2911271

[B87] Maurel C. (1997). Aquaporins and water permeability of plant membranes. *Annu. Rev. Plant Biol.* 48 399–429. 10.1146/annurev.arplant.48.1.399 15012269

[B88] McLean E. H. Ludwig M. Grierson P. F. (2011). Root hydraulic conductance and aquaporin abundance respond rapidly to partial root-zone drying events in a riparian *Melaleuca* species. *New Phytol.* 192 664–675. 10.1111/j.1469-8137.2011.03834.x 21848988

[B89] Medrano H. Escalona J. Bota J. Gulias J. Flexas J. (2002). Regulation of photosynthesis of C3 plants in response to progressive drought: stomatal conductance as a reference parameter. *Ann. Bot.* 89 895–905. 10.1093/aob/mcf079 12102515 PMC4233802

[B90] Meng D. Fricke W. (2017). Changes in root hydraulic conductivity facilitate the overall hydraulic response of rice (*Oryza sativa* L.) cultivars to salt and osmotic stress. *Plant Physiol. Biochem.* 113 64–77. 10.1016/j.plaphy.2017.02.001 28189051

[B91] Mihailović N. Jelić G. Filipović R. Djurdjević M. Dželetović Ž. (1992). Effect of nitrogen form on maize response to drought stress. *Plant Soil* 144 191–197. 10.1007/BF00012875

[B92] Miyazawa S. -I. Yoshimura S. Shinzaki Y. Maeshima M. Miyake C. (2008). Deactivation of aquaporins decreases internal conductance to CO2 diffusion in tobacco leaves grown under long-term drought. *Funct. Plant Biol.* 35 553–564. 10.1071/FP08117 32688811

[B93] Mizokami Y. Noguchi K. Kojima M. Sakakibara H. Terashima I. (2015). Mesophyll conductance decreases in the wild type but not in an ABA-deficient mutant (*aba1*) of *Nicotiana plumbaginifolia* under drought conditions. *Plant Cell Environ.* 38 388–398. 10.1111/pce.12394 24995523

[B94] Morgan J. M. (1984). Osmoregulation and water stress in higher plants. *Annu. Rev. Plant Physiol.* 35 299–319. 10.1146/annurev.pp.35.060184.001503

[B95] Mylona P. Pawlowski K. Bisseling T. (1995). Symbiotic nitrogen fixation. *Plant Cell* 7 869–885. 10.1105/tpc.7.7.869 12242391 PMC160880

[B96] Nardini A. Salleo S. (2005). Water stress-induced modifications of leaf hydraulic architecture in sunflower: co-ordination with gas exchange. *J. Exp. Bot.* 56 3093–3101. 10.1093/jxb/eri306 16246857

[B97] Niemietz C. M. Tyerman S. D. (2000). Channel-mediated permeation of ammonia gas through the peribacteroid membrane of soybean nodules. *FEBS Lett.* 465 110–114. 10.1016/S0014-5793(99)01729-9 10631315

[B98] North G. Martre P. Nobel P. (2004). Aquaporins account for variations in hydraulic conductance for metabolically active root regions of *Agave deserti* in wet, dry, and rewetted soil. *Plant Cell Environ.* 27 219–228. 10.1111/j.1365-3040.2003.01137.x

[B99] Parent B. Hachez C. Redondo E. Simonneau T. Chaumont F. Tardieu F. (2009). Drought and abscisic acid effects on aquaporin content translate into changes in hydraulic conductivity and leaf growth rate: a trans-scale approach. *Plant Physiol.* 149 2000–2012. 10.1104/pp.108.130682 19211703 PMC2663730

[B100] Park W. Scheffler B. E. Bauer P. J. Campbell B. T. (2010). Identification of the family of aquaporin genes and their expression in upland cotton (*Gossypium hirsutum* L.). *BMC Plant Biol.* 10:142. 10.1186/1471-2229-10-142 20626869 PMC3095289

[B101] Parry M. Canziani O. Palutikof J. Van Der Linden P. J. Hanson C. E. (2007). *Climate Change 2007: Impacts, Adaptation and Vulnerability.* Cambridge: Cambridge University Press.

[B102] Peng Y. Lin W. Cai W. Arora R. (2007). Overexpression of a *Panax ginseng* tonoplast aquaporin alters salt tolerance, drought tolerance and cold acclimation ability in transgenic Arabidopsis plants. *Planta* 226 729–740. 10.1007/s00425-007-0520-4 17443343

[B103] Perez-Martin A. Michelazzo C. Torres-Ruiz J. M. Flexas J. Fernandez J. E. Sebastiani L. (2014). Regulation of photosynthesis and stomatal and mesophyll conductance under water stress and recovery in olive trees: correlation with gene expression of carbonic anhydrase and aquaporins. *J. Exp. Bot.* 65 3143–3156. 10.1093/jxb/eru160 24799563 PMC4071832

[B104] Pinheiro H. A. Damatta F. M. Chaves A. R. Loureiro M. E. Ducatti C. (2005). Drought tolerance is associated with rooting depth and stomatal control of water use in clones of *Coffea canephora*. *Ann. Bot.* 96 101–108. 10.1093/aob/mci154 15888500 PMC4246813

[B105] Postaire O. Tournaire-Roux C. Grondin A. Boursiac Y. Morillon R. Schaffner A. R. (2010). A PIP1 aquaporin contributes to hydrostatic pressure-induced water transport in both the root and rosette of Arabidopsis. *Plant Physiol.* 152 1418–1430. 10.1104/pp.109.145326 20034965 PMC2832249

[B106] Pou A. Medrano H. Flexas J. Tyerman S. D. (2013). A putative role for TIP and PIP aquaporins in dynamics of leaf hydraulic and stomatal conductances in grapevine under water stress and re-watering. *Plant Cell Environ.* 36 828–843. 10.1111/pce.12019 23046275

[B107] Qian Z. J. Song J. J. Chaumont F. Ye Q. (2015). Differential responses of plasma membrane aquaporins in mediating water transport of cucumber seedlings under osmotic and salt stresses. *Plant Cell Environ.* 38 461–473. 10.1111/pce.12319 24601940

[B108] Ranathunge K. Kotula L. Steudle E. Lafitte R. (2004). Water permeability and reflection coefficient of the outer part of young rice roots are differently affected by closure of water channels (aquaporins) or blockage of apoplastic pores. *J. Exp. Bot.* 55 433–447. 10.1093/jxb/erh041 14739266

[B109] Ranathunge K. Schreiber L. Bi Y.-M. Rothstein S. J. (2016). Ammonium-induced architectural and anatomical changes with altered suberin and lignin levels significantly change water and solute permeabilities of rice (*Oryza sativa* L.) roots. *Planta* 243 231–249. 10.1007/s00425-015-2406-1 26384983

[B110] Ranathunge K. Steudle E. Lafitte R. (2003). Control of water uptake by rice (*Oryza sativa* L.): role of the outer part of the root. *Planta* 217 193–205. 12783327 10.1007/s00425-003-0984-9

[B111] Ren B. Wang M. Chen Y. Sun G. Li Y. Shen Q. (2015). Water absorption is affected by the nitrogen supply to rice plants. *Plant Soil* 396 397–410. 10.1007/s11104-015-2603-5

[B112] Rivers R. L. Dean R. M. Chandy G. Hall J. E. Roberts D. M. Zeidel M. L. (1997). Functional analysis of nodulin 26, an aquaporin in soybean root nodule symbiosomes. *J. Biol. Chem.* 272 16256–16261. 10.1074/jbc.272.26.16256 9195927

[B113] Roberts D. M. Routray P. (2017). “The nodulin 26 intrinsic protein subfamily,” in *Plant Aquaporins*, eds Chaumont F. Tyerman S. (Cham: Springer), 267–296.

[B114] Rodrigues O. Reshetnyak G. Grondin A. Saijo Y. Leonhardt N. Maurel C. (2017). Aquaporins facilitate hydrogen peroxide entry into guard cells to mediate ABA-and pathogen-triggered stomatal closure. *Proc. Natl. Acad. Sci. U.S.A.* 114 9200–9205. 10.1073/pnas.1704754114 28784763 PMC5576802

[B115] Roth L. Stacey G. (1989). Bacterium release into host cells of nitrogen-fixing soybean nodules: the symbiosome membrane comes from three sources. *Eur. J. Cell Biol.* 49 13–23. 2759097

[B116] Sack L. Holbrook N. M. (2006). Leaf hydraulics. *Annu. Rev. Plant Biol.* 57 361–381. 10.1146/annurev.arplant.56.032604.144141 16669766

[B117] Sade N. Moshelion M. (2017). “Plant aquaporins and abiotic stress,” in *Plant Aquaporins*, eds Chaumont F. Tyerman S. D. (Berlin: Springer), 185–206.

[B118] Sade N. Shatil-Cohen A. Moshelion M. (2015). Bundle-sheath aquaporins play a role in controlling Arabidopsis leaf hydraulic conductivity. *Plant Signal. Behav.* 10:e1017177. 10.1080/15592324.2015.1017177 26039476 PMC4623468

[B119] Sade N. Vinocur B. J. Diber A. Shatil A. Ronen G. Nissan H. (2009). Improving plant stress tolerance and yield production: is the tonoplast aquaporin SlTIP2; 2 a key to isohydric to anisohydric conversion? *New Phytol.* 181 651–661. 10.1111/j.1469-8137.2008.02689.x 19054338

[B120] Sakurai J. Ishikawa F. Yamaguchi T. Uemura M. Maeshima M. (2005). Identification of 33 rice aquaporin genes and analysis of their expression and function. *Plant Cell Physiol.* 46 1568–1577. 10.1093/pcp/pci172 16033806

[B121] Schulze E. (1986). Carbon dioxide and water vapor exchange in response to drought in the atmosphere and in the soil. *Annu. Rev. Plant Physiol.* 37 247–274. 10.1146/annurev.pp.37.060186.001335

[B122] Schwab K. Schreiber U. Heber U. (1989). Response of photosynthesis and respiration of resurrection plants to desiccation and rehydration. *Planta* 177 217–227. 10.1007/BF00392810 24212344

[B123] Scoffoni C. Chatelet D. S. Pasquet-Kok J. Rawls M. Donoghue M. J. Edwards E. J. (2016). Hydraulic basis for the evolution of photosynthetic productivity. *Nat. Plants* 2:16072. 10.1038/nplants.2016.72 27255836

[B124] Scoffoni C. Mckown A. D. Rawls M. Sack L. (2011a). Dynamics of leaf hydraulic conductance with water status: quantification and analysis of species differences under steady state. *J. Exp. Bot.* 63 643–658. 10.1093/jxb/err270 22016424 PMC3254676

[B125] Scoffoni C. Rawls M. Mckown A. Cochard H. Sack L. (2011b). Decline of leaf hydraulic conductance with dehydration: relationship to leaf size and venation architecture. *Plant Physiol.* 156 832–843. 10.1104/pp.111.173856 21511989 PMC3177279

[B126] Secchi F. Zwieniecki M. A. (2014). Down-regulation of plasma intrinsic protein1 aquaporin in poplar trees is detrimental to recovery from embolism. *Plant Physiol.* 164 1789–1799. 10.1104/pp.114.237511 24572173 PMC3982741

[B127] Seki M. Umezawa T. Urano K. Shinozaki K. (2007). Regulatory metabolic networks in drought stress responses. *Curr. Opin. Plant Biol.* 10 296–302. 10.1016/j.pbi.2007.04.014 17468040

[B128] Sharp R. E. (2002). Interaction with ethylene: changing views on the role of abscisic acid in root and shoot growth responses to water stress. *Plant Cell Environ.* 25 211–222. 10.1046/j.1365-3040.2002.00798.x 11841664

[B129] Shatil-Cohen A. Attia Z. Moshelion M. (2011). Bundle-sheath cell regulation of xylem-mesophyll water transport via aquaporins under drought stress: a target of xylem-borne ABA? *Plant J.* 67 72–80. 10.1111/j.1365-313X.2011.04576.x 21401747

[B130] Siefritz F. Tyree M. T. Lovisolo C. Schubert A. Kaldenhoff R. (2002). PIP1 plasma membrane aquaporins in tobacco from cellular effects to function in plants. *Plant Cell* 14 869–876. 10.1105/tpc.000901 11971141 PMC150688

[B131] Skelton R. P. Brodribb T. J. Mcadam S. A. Mitchell P. J. (2017). Gas exchange recovery following natural drought is rapid unless limited by loss of leaf hydraulic conductance: evidence from an evergreen woodland. *New Phytol.* 215 1399–1412. 10.1111/nph.14652 28620915

[B132] Skirycz A. Inzé D. (2010). More from less: plant growth under limited water. *Curr. Opin. Biotechnol.* 21 197–203. 10.1016/j.copbio.2010.03.002 20363612

[B133] Smith S. E. Smith F. A. (2011). Roles of arbuscular mycorrhizas in plant nutrition and growth: new paradigms from cellular to ecosystem scales. *Annu. Rev. Plant Biol.* 62 227–250. 10.1146/annurev-arplant-042110-103846 21391813

[B134] Soto G. Fox R. Ayub N. Alleva K. Guaimas F. Erijman E. J. (2010). TIP5; 1 is an aquaporin specifically targeted to pollen mitochondria and is probably involved in nitrogen remobilization in *Arabidopsis thaliana*. *Plant J.* 64 1038–1047. 10.1111/j.1365-313X.2010.04395.x 21143683

[B135] Sreedharan S. Shekhawat U. K. Ganapathi T. R. (2013). Transgenic banana plants overexpressing a native plasma membrane aquaporin MusaPIP1; 2 display high tolerance levels to different abiotic stresses. *Plant Biotechnol. J.* 11 942–952. 10.1111/pbi.12086 23745761

[B136] Steudle E. (2000a). Water uptake by plant roots: an integration of views. *Plant Soil* 226 45–56. 10.1023/A:1026439226716

[B137] Steudle E. (2000b). Water uptake by roots: effects of water deficit. *J. Exp. Bot.* 51 1531–1542. 10.1093/jexbot/51.350.1531 11006304

[B138] Steudle E. Peterson C. A. (1998). How does water get through roots? *J. Exp. Bot.* 49 775–788. 10.1093/jxb/49.322.775

[B139] Tabassum M. A. Zhu G. Hafeez A. Wahid M. A. Shaban M. Li Y. (2016). Influence of leaf vein density and thickness on hydraulic conductance and photosynthesis in rice (*Oryza sativa* L.) during water stress. *Sci. Rep.* 6:36894. 10.1038/srep36894 27848980 PMC5111110

[B140] Takano J. Miwa K. Fujiwara T. (2008). Boron transport mechanisms: collaboration of channels and transporters. *Trends Plant Sci.* 13 451–457. 10.1016/j.tplants.2008.05.007 18603465

[B141] Tang A. C. Kawamitsu Y. Kanechi M. Boyer J. S. (2002). Photosynthetic oxygen evolution at low water potential in leaf discs lacking an epidermis. *Ann. Bot.* 89 861–870. 10.1093/aob/mcf081 12102512 PMC4233803

[B142] Tezara W. Mitchell V. Driscoll S. Lawlor D. (1999). Water stress inhibits plant photosynthesis by decreasing coupling factor and ATP. *Nature* 401 914–917. 10.1038/44842

[B143] Tyerman S. D. Wignes J. A. Kaiser B. N. (2017). “Root Hydraulic and Aquaporin Responses to N Availability,” in *Plant Aquaporins*, eds Chaumont F. Tyerman S. (Cham: Springer), 207–236.

[B144] Udvardi M. Poole P. S. (2013). Transport and metabolism in legume-rhizobia symbioses. *Annu. Rev. Plant Biol.* 64 781–805. 10.1146/annurev-arplant-050312-120235 23451778

[B145] Uehlein N. Fileschi K. Eckert M. Bienert G. P. Bertl A. Kaldenhoff R. (2007). Arbuscular mycorrhizal symbiosis and plant aquaporin expression. *Phytochemistry* 68 122–129. 10.1016/j.phytochem.2006.09.033 17109903

[B146] Uehlein N. Kai L. Kaldenhoff R. (2017). “Plant Aquaporins and CO2,” in *Plant Aquaporins*, eds Chaumont F. Tyerman S. (Cham: Springer), 255–265. 10.1007/978-3-319-49395-4_12

[B147] Vandeleur R. K. Mayo G. Shelden M. C. Gilliham M. Kaiser B. N. Tyerman S. D. (2009). The role of plasma membrane intrinsic protein aquaporins in water transport through roots: diurnal and drought stress responses reveal different strategies between isohydric and anisohydric cultivars of grapevine. *Plant Physiol.* 149 445–460. 10.1104/pp.108.128645 18987216 PMC2613730

[B148] Vandeleur R. K. Sullivan W. Athman A. Jordans C. Gilliham M. Kaiser B. N. (2014). Rapid shoot-to-root signalling regulates root hydraulic conductance via aquaporins. *Plant Cell Environ.* 37 520–538. 10.1111/pce.12175 23926961

[B149] Vilagrosa A. Chirino E. Peguero-Pina J. -J. Barigah T. S. Cochard H. Gil-Pelegrin E. (2012). “Xylem cavitation and embolism in plants living in water-limited ecosystems,” in *Plant Responses to Drought Stress*, ed. Aroca R. (Berlin: Springer), 63–109.

[B150] Wang L. Li Q.-T. Lei Q. Feng C. Zheng X. Zhou F. (2017). Ectopically expressing *MdPIP1; 3*, an aquaporin gene, increased fruit size and enhanced drought tolerance of transgenic tomatoes. *BMC Plant Biol.* 17:246. 10.1186/s12870-017-1212-2 29258418 PMC5735821

[B151] Wang Y. Li R. Li D. Jia X. Zhou D. Li J. (2017). NIP1; 2 is a plasma membrane-localized transporter mediating aluminum uptake, translocation, and tolerance in Arabidopsis. *Proc. Natl. Acad. Sci. U.S.A.* 114 5047–5052. 10.1073/pnas.1618557114 28439024 PMC5441725

[B152] Wang M. Ding L. Gao L. Li Y. Shen Q. Guo S. (2016). The Interactions of aquaporins and mineral nutrients in higher plants. *Int. J. Mol. Sci.* 17:E1229. 10.3390/ijms17081229 27483251 PMC5000627

[B153] Wang M. Shen Q. R. Xu G. H. Guo S. W. (2014). New insight into the strategy for nitrogen metabolism in plant cells. *Int. Rev. Cell Mol. Biol.* 310 1–37. 10.1016/B978-0-12-800180-6.00001-3 24725423

[B154] Weaver C. D. Crombie B. Stacey G. Roberts D. M. (1991). Calcium-dependent phosphorylation of symbiosome membrane proteins from nitrogen-fixing soybean nodules: evidence for phosphorylation of nodulin-26. *Plant Physiol.* 95 222–227. 10.1104/pp.95.1.222 16667955 PMC1077509

[B155] Wilkinson S. Bacon M. A. Davies W. J. (2007). Nitrate signalling to stomata and growing leaves: interactions with soil drying, ABA, and xylem sap pH in maize. *J. Exp. Bot.* 58 1705–1716. 10.1093/jxb/erm021 17374875

[B156] Witte C.-P. (2011). Urea metabolism in plants. *Plant Sci.* 180 431–438. 10.1016/j.plantsci.2010.11.010 21421389

[B157] Xiong D. Flexas J. Yu T. Peng S. Huang J. (2017). Leaf anatomy mediates coordination of leaf hydraulic conductance and mesophyll conductance to CO2 in Oryza. *New Phytol.* 213 572–583. 10.1111/nph.14186 27653809

[B158] Xu G. Fan X. Miller A. J. (2012). Plant nitrogen assimilation and use efficiency. *Annu. Rev. Plant Biol.* 63 153–182. 10.1146/annurev-arplant-042811-105532 22224450

[B159] Xu Y. Hu W. Liu J. Zhang J. Jia C. Miao H. (2014). A banana aquaporin gene, *MaPIP1; 1*, is involved in tolerance to drought and salt stresses. *BMC Plant Biol.* 14:59. 10.1186/1471-2229-14-59 24606771 PMC4015420

[B160] Xu Z. Zhou G. Shimizu H. (2009). Are plant growth and photosynthesis limited by pre-drought following rewatering in grass? *J. Exp. Bot.* 60 3737–3749. 10.1093/jxb/erp216 19596698 PMC2736889

[B161] Xu Z. Zhou G. Shimizu H. (2010). Plant responses to drought and rewatering. *Plant Signal. Behav.* 5 649–654. 10.4161/psb.5.6.11398 20404516 PMC3001553

[B162] Yang H. Menz J. Häussermann I. Benz M. Fujiwara T. Ludewig U. (2015). High and low affinity urea root uptake: involvement of NIP5; 1. *Plant Cell Physiol.* 56 1588–1597. 10.1093/pcp/pcv067 25957355

[B163] Yang X. X. Li Y. Ren B. B. Ding L. Gao C. M. Shen Q. R. (2012). Drought-induced root aerenchyma formation restricts water uptake in rice seedlings supplied with nitrate. *Plant Cell Physiol.* 53 495–504. 10.1093/pcp/pcs003 22257489

[B164] Yu G. Li J. Sun X. Zhang X. Liu J. Pan H. (2015). Overexpression of AcNIP5; 1, a novel nodulin-like intrinsic protein from halophyte *Atriplex canescens*, enhances sensitivity to salinity and improves drought tolerance in Arabidopsis. *Plant Mol. Biol. Rep.* 33 1864–1875. 10.1007/s11105-015-0881-y

[B165] Yu Q. Hu Y. Li J. Wu Q. Lin Z. (2005). Sense and antisense expression of plasma membrane aquaporin BnPIP1 from *Brassica napus* in tobacco and its effects on plant drought resistance. *Plant Sci.* 169 647–656. 10.1016/j.plantsci.2005.04.013

[B166] Zhang D. Y. Ali Z. Wang C. B. Xu L. Yi J. X. Xu Z. L. (2013). Genome-wide sequence characterization and expression analysis of major intrinsic proteins in soybean (*Glycine max* L.). *PLoS One* 8:e56312. 10.1371/journal.pone.0056312 23437113 PMC3577755

[B167] Zhang D.-Y. Kumar M. Xu L. Wan Q. Huang Y.-H. Xu Z.-L. (2017). Genome-wide identification of major intrinsic proteins in *Glycine soja* and characterization of GmTIP2; 1 function under salt and water stress. *Sci. Rep.* 7:4106. 10.1038/s41598-017-04253-z 28646139 PMC5482899

[B168] Zhang J. Jia W. Yang J. Ismail A. M. (2006). Role of ABA in integrating plant responses to drought and salt stresses. *Field Crops Res.* 97 111–119. 10.1016/j.fcr.2005.08.018 26093896

[B169] Zhang L. Yan J. Vatamaniuk O. K. Du X. (2016). CsNIP2; 1 is a plasma membrane transporter from *Cucumis sativus* that facilitates urea uptake when expressed in *Saccharomyces cerevisiae* and *Arabidopsis thaliana*. *Plant Cell Physiol.* 57 616–629. 10.1093/pcp/pcw018 26858284

[B170] Zhang X. Zhang L. Dong F. Gao J. Galbraith D. W. Song C.-P. (2001). Hydrogen peroxide is involved in abscisic acid-induced stomatal closure in *Vicia faba*. *Plant Physiol.* 126 1438–1448. 10.1104/pp.126.4.1438 11500543 PMC117144

[B171] Zhang Y. Wang Z. Chai T. Wen Z. Zhang H. (2008). Indian mustard aquaporin improves drought and heavy-metal resistance in tobacco. *Mol. Biotechnol.* 40 280–292. 10.1007/s12033-008-9084-1 18622723

[B172] Zhou Y. Lam H. M. Zhang J. (2007). Inhibition of photosynthesis and energy dissipation induced by water and high light stresses in rice. *J. Exp. Bot.* 58 1207–1217. 10.1093/jxb/erl291 17283375

[B173] Zhuang L. Liu M. Yuan X. Yang Z. Huang B. (2015). Physiological effects of aquaporin in regulating drought tolerance through overexpressing of *Festuca arundinacea* aquaporin gene FaPIP2; 1. *J. Am. Soc. Hortic. Sci.* 140 404–412.

